# Editorial: Multi-omics interrogation of tumor-associated macrophages: paving the way for next-generation cancer immunotherapies

**DOI:** 10.3389/fimmu.2026.1961613

**Published:** 2026-08-25

**Authors:** Shengshan Xu, Ke-Jie He, Lin Zhang, Qian Guo

**Affiliations:** 1 Department of Thoracic Surgery, Jiangmen Central Hospital, Jiangmen, Guangdong, China; 2 The Quzhou Affiliated Hospital of Wenzhou Medical University, Quzhou People’s Hospital, Quzhou, Zhejiang, China; 3 The School of Public Health and Preventive Medicine, Monash University, Melbourne, Australia; 4 Zhongnan Hospital of Wuhan University, Wuhan, China

**Keywords:** cancer immunotherapy, multi-omics, prognostic biomarkers, tumor microenvironment, tumor-associated macrophages

## Introduction

The tumor microenvironment (TME) is a highly complex and dynamic ecosystem that dictates cancer progression, metastasis, and therapeutic response. Within this milieu, tumor-associated macrophages (TAMs) have emerged as central orchestrators of immunosuppression. While traditionally oversimplified into a dichotomy of anti-tumoral (M1) and pro-tumoral (M2) phenotypes, the advent of high-resolution multi-omics technologies—including single-cell RNA sequencing (scRNA-seq), spatial transcriptomics, and spatial proteomics—has revealed staggering heterogeneity and plasticity within the TAM population.

This Research Topic, “Multi-Omics Interrogation of Tumor-Associated Macrophages: Paving the Way for Next-Generation Cancer Immunotherapies,” compiles 15 articles that collectively advance our understanding of TAM biology. By dissecting TAM-tumor crosstalk, mapping immune-evasion mechanisms, and identifying novel prognostic signatures and therapeutic targets, these contributions provide a crucial roadmap for overcoming myeloid-driven resistance to current immunotherapies.

## Decoding TAM heterogeneity and function across malignancies

A significant portion of this Research Topic is dedicated to comprehensively reviewing the multifaceted roles of TAMs across various solid tumors. Zhu et al. provide a foundational overview of TAM functional diversity, outlining how these cells interact within the TME to drive tumor advancement and summarizing current immunotherapeutic strategies aimed at TAM depletion and reprogramming.

In hormone-driven and gender-specific cancers, TAMs pose unique challenges. Wu et al. detail how TAMs facilitate angiogenesis and metastasis in Triple-Negative Breast Cancer via IL-10 and TGF-β signaling, emphasizing the clinical promise of blocking the CD47-SIRPα phagocytosis checkpoint. Similarly, Li et al. (M. Li) explore the ontogeny of TAMs in ovarian cancer, highlighting their critical involvement in driving platinum resistance through exosomal signaling and metabolic reprogramming. In the context of prostate cancer, Chen et al. present a multi-omics perspective on TAM plasticity during androgen deprivation therapy and PARP inhibition. They propose a novel Myeloid Lymphatic Composite Score to assist in timing therapeutic combinations, capitalizing on the transition of TAMs to immunosuppressive states under treatment pressure.

In the thoracic and gastrointestinal tracts, Hao et al. review the mechanisms by which M2-like TAMs promote immune evasion and chemoresistance in non-small cell lung cancer, noting the paradoxical association of TAM infiltration with PD-1/PD-L1 blockade responses in certain cohorts. Wang et al. discuss the role of TAMs in gastric cancer, focusing on their involvement in lymphovascular invasion and evaluating targeted interventions such as NF-κB inhibitors.

Finally, the unique challenges of the central nervous system are addressed. Ma et al. explore the diversity of TAMs in brain metastases, moving beyond the M1/M2 paradigm to discuss lipid-associated and pro-angiogenic subpopulations that disrupt the blood-brain barrier. In primary diffuse gliomas, Zhang et al. review ligand-receptor multi-omics, identifying critical perivascular communication axes and appraising translational opportunities for innate-adaptive combination therapies.

## Advancing prognosis and stratification via multi-omics frameworks

The integration of multi-omics data with machine learning has enabled the construction of robust, TAM-centric prognostic models. Shi et al. utilized a Scissor-guided single-cell framework to map clinical phenotypes onto LUAD scRNA-seq data. They developed the Scissor-Associated Macrophage Risk Score, demonstrating that high-risk scores correlate with higher mutation burdens and immune evasion, while identifying TIMP1 as a key regulatory gene in macrophage-associated tumor progression.

In breast cancer, Pei et al. leveraged bulk and scRNA-seq data to construct a prognostic model based on macrophage-related genes. Their model successfully stratified patients by overall survival and immunotherapy response, while concurrently identifying HAGHL as a novel oncogenic driver whose knockdown inhibits BRCA cell proliferation and invasion.

Expanding to a pan-cancer perspective, Li et al. (S. Li) integrated scRNA-seq and spatial transcriptomics to investigate the relationship between TAMs and T cell exhaustion. They identified a specific subset of STMN2+ TAMs associated with poor immunotherapy responses. Notably, their functional assays in neuroblastoma revealed that silencing BCAP31 on TAMs can drive CD8+ T cells toward an active effector state, uncovering a novel target for reversing immune exhaustion.

## Uncovering novel targets and environmental triggers

Original research within this Research Topic also pinpoints specific molecular drivers of TAM activation and immune exclusion. In colorectal cancer (CRC), Guan et al. utilized non-negative matrix factorization and WGCNA on multi-cohort scRNA-seq data to identify GPR35 as a master regulator of M2 macrophage activation. High GPR35 expression orchestrated a “cold” immune-excluded microenvironment, positioning it as a promising target to sensitize CRC to immune checkpoint blockade. In gastric cancer, Li et al. applied machine learning to multi-omics data, identifying EFNA3 as a core biomarker associated with the invasive phenotype, immunosuppression, and malignant cell proliferation both *in vitro* and *in vivo*.

Using an innovative multi-sample Mendelian randomization (MR) approach combined with single-cell virtual knockout modeling, Feng et al. identified plasma prefoldin subunit 2 (PFDN2) as a protective factor in head and neck squamous cell carcinoma. They demonstrated that the PFDN2-CD64 interaction restricts monocyte-driven inflammatory microenvironments, offering a unique systemic perspective on local tumor immunity.

Lastly, Gao et al. provide compelling real-world evidence bridging occupational health and tumor immunology. Analyzing data from industrial workers alongside experimental models, they demonstrated that occupational silica exposure induces macrophage-driven inflammatory signaling (via IL-6 and NF-κB). This systemic inflammation remodels the TME and promotes early elevation of the tumor marker CEA, highlighting a crucial link between environmental exposure and immune-related cancer risk.

## Conclusion

The articles curated in this Research Topic unequivocally demonstrate that moving beyond the simplified M1/M2 classification is essential for the future of immuno-oncology. By leveraging multi-omics technologies, the contributing authors have uncovered spatially organized, transcriptionally distinct TAM subpopulations that dynamically respond to therapies and environmental exposures. Furthermore, the identification of novel biomarkers and therapeutic targets (GPR35, EFNA3, PFDN2, BCAP31, TIMP1, HAGHL) provides actionable avenues for preclinical and clinical development. As we look to the future, the integration of these high-resolution, TAM-informed metrics into clinical trial design will be paramount for converting resistant, immunosuppressive tumors into responsive ecosystems, ultimately paving the way for next-generation precision immunotherapies.

